# Integrative analysis of seed morphology, geographic origin, and genetic structure in *Medicago* with implications for breeding and conservation

**DOI:** 10.1186/s12870-025-06304-4

**Published:** 2025-03-03

**Authors:** Seunghyun Lim, Sunchung Park, Insuck Baek, Jacob Botkin, Jae Hee Jang, Seok Min Hong, Brian M. Irish, Moon S. Kim, Lyndel W. Meinhardt, Shaun J. Curtin, Ezekiel Ahn

**Affiliations:** 1 Sustainable Perennial Crops Laboratory, Agricultural Research Service, Department of Agriculture, Beltsville, MD 20705 USA; 2 Environmental Microbial and Food Safety Laboratory, Agricultural Research Service, Department of Agriculture, Beltsville, MD 20705 USA; 3 Department of Plant Pathology, University of Minnesota, St. Paul, MN 55108 USA; 4 Department of Civil Urban Earth and Environmental Engineering, Ulsan National Institute of Science and Technology, UNIST-gil 50, Ulsan, 44919 Republic of Korea; 5 Plant Germplasm Introduction and Testing Research Unit, Department of Agriculture, Prosser, WA 99164 USA; 6 Department of Agronomy and Plant Genetics, University of Minnesota, St. Paul, MN 55108 USA; 7 Plant Science Research Unit, Department of Agriculture- Agricultural Research Service, St. Paul, MN 55108 USA; 8 Center for Genome Engineering, University of Minnesota, St. Paul, MN 55108 USA

**Keywords:** Seed morhology, Medicago, Geographic origin, Genetic diversity, Machine learning, GWAS, Local adaptation, Alfalfa

## Abstract

**Background:**

Seed morphology and color are critical agronomic traits in *Medicago* spp., reflecting adaptations to diverse environments and influencing seedling establishment and vigor. Understanding the interplay between seed traits, geographic origin, and genetic diversity is crucial for effective germplasm conservation and breeding. This study presents a comprehensive analysis of these factors in a diverse collection of *Medicago* accessions, leveraging machine learning to illuminate these complex relationships.

**Results:**

We analyzed seed size, shape, and color data from 318 *Medicago* accessions representing 29 species/subspecies from 31 countries. Machine learning models, including Neural Boost, Bootstrap Forest, and Support Vector Machines, effectively classified accessions based on seed traits and geographic origin, achieving up to 80% accuracy. Seed size was accurately predicted (R-squared > 0.80) using a combination of species, geographic origin, and shape descriptors. Hierarchical clustering of 189 *M. sativa* accessions based on 8,565 SNP markers revealed 20 distinct genetic clusters, indicating substantial population structure. A machine learning-based genome-wide association (GWA) analysis identified SNPs on chromosomes 1, 6, and 8 with high importance for predicting geographic origin. Notably, the most significant SNPs were located in or near genes involved in stress response and genome stability, suggesting their potential role in local adaptation. Finally, we successfully imputed missing *M. sativa* SNP genotypes using multiple machine learning approaches, achieving over 70% accuracy overall and over 80% for individual nucleotides (A, T, C, G), enhancing the utility of genomic datasets with missing data.

**Conclusions:**

Our integrated analysis of phenotypic, genetic, and geographic data, coupled with a machine learning-based GWAS approach, provides valuable insights into the diverse patterns within *Medicago* spp. We demonstrate the power of machine learning for germplasm characterization, trait prediction, and imputation of missing genomic data. These findings have significant implications for seed trait improvement, germplasm management, and understanding adaptation in *Medicago* and other diverse crop species. The identified candidate genes associated with geographic origin provide a foundation for future investigations into the functional mechanisms of local adaptation. Furthermore, our imputation method offers a valuable data for maximizing the utility of genomic resources in *Medicago* and other species.

## Introduction

The genus *Medicago* encompasses a diverse group of legumes, including the economically important forage crop alfalfa (*Medicago sativa* L.) and other species with significant ecological and agricultural value [1]. These species play vital roles in various ecosystems by contributing to nitrogen fixation [2] and soil improvement [3], as well as providing forage for livestock [4]. Understanding the patterns of phenotypic and genetic diversity within *Medicago* spp. is crucial for successful conservation of germplasm resources and breeding programs aimed at developing improved varieties with enhanced agronomic traits [5]. Seed characteristics such as size, shape, and color represent a particularly important suite of traits in *Medicago* spp. and other legumes [6]. When it comes to size and shape, alfalfa seeds are rather small and oblong; a study conducted in 2023 found that the seeds of 20 alfalfa genotypes had an average length of 1.265 mm, an average width of 0.732 mm, an average surface area of 0.761 mm^2^, and a mean sphericity value of 0.334 [7]. These physical traits are not only closely linked to seedling vigor (a complex trait that determines the ability of seeds to germinate and establish seedlings rapidly) [8–11], emergence success [12], and overall plant fitness [13], but they also often reflect adaptation to specific environmental conditions and dispersal mechanisms [14]. Consequently, exploring the variation in seed morphology and its relationship to geographic origin and genetic diversity can provide valuable insights into the evolutionary history and adaptive potential of *Medicago* spp.

Previous research has emphasized the considerable phenotypic and genetic diversity within the *Medicago* genus [15–17]. A study focusing on seed morphology revealed significant variations in seed size, shape, and color among different *Medicago* spp. and even among populations within the same species [6]. This variation could be linked to factors such as geographic origin [18–20], ploidy level [21], and adaptation to specific environmental conditions [22]. Furthermore, genetic analyses using various molecular markers have provided insights on the population structure, genetic relationships, and evolutionary history of *Medicago* species [22, 23]. Previous studies on *Medicago* have uncovered a strong geographic component to genetic differentiation, implying that isolation by both distance and adaptation to local environments has a key role in shaping species diversity. For example, a study on *M. laciniata* and *M. truncatula* in Tunisia found substantial genetic differentiation among populations, with 5.85% and 11.27% of the total quantitative genetic variability explained by the site of origin, respectively [24]. This differentiation was linked to both geographic distance and adaptation to local environmental conditions, accentuating the interplay between these factors in shaping *Medicago* diversity [24]. Moreover, an analysis of *M. ciliaris* populations in Tunisia revealed that while overall genetic differentiation was low, significant correlations between phenotypic traits and eco-geographical factors, such as soil texture and altitude, point to local adaptation playing a crucial role in shaping diversity within this species [25]. Despite the growing availability of high-density SNP genotype data and detailed phenotypic information for *Medicago* spp [26, 27], including seed morphology [6], research integrating such data with geographic origin and advanced machine learning techniques remains limited. This study aims to address this gap by investigating the complex relationships between seed morphology, color, geographic origin, and genetic diversity in a large and diverse collection of *Medicago* accessions. Understanding these relationships is crucial for effective germplasm conservation and breeding programs. Specifically, we aim to answer the following research questions: (1) How does seed morphology and color vary across different *Medicago* spp. and geographic origins? (2) Can machine learning models effectively classify *Medicago* spp. based on seed traits and geographic origin? (3) Can machine learning models be used to predict seed size in *Medicago* spp.? (4) What is the genetic structure of the *M. sativa* collection, and do SNP-based clusters correlate with phenotypic or geographic patterns? (5) Can we identify genomic regions associated with geographic origin using a machine learning-based GWA approach?

## Materials and methods

### Overview of approach

This study employed a multi-faceted approach combining phenotypic data with genomic analysis and machine learning. We utilized publicly available data from a diverse collection of 319 *Medicago* accessions, representing 29 species/subspecies and originating from 31 countries across different continents [6, 28]. Publicly available Genotyping-by-Sequencing (GBS) data was used to generate a high-density SNP dataset for 189 *M. sativa* accessions, which was then applied towards genetic clustering analysis and a machine learning-based GWA study to identify SNPs associated with geographic origin. We then evaluated the performance of various machine learning models in classifying accessions based on seed traits and geographic origin, and predicting seed size. Additionally, we employed a machine learning-based approach for imputing missing SNP genotypes by using a subset of loci with complete data to predict missing genotypes in other accessions. This integrated analysis provides a comprehensive assessment of phenotypic and genetic diversity in *Medicago* spp. and highlights the potential of machine learning for germplasm characterization and genomic analysis.

### Quantification of genetic distance and correlation with seed morphological traits

Seed morphological trait data were obtained from our previous study [6], in which Botkin et al. established the utility of image analysis and machine learning for characterizing seed morphology in *Medicago* spp. and identified preliminary associations between seed traits and genetic markers [6]. The current study builds upon these findings by integrating geographic data and employing advanced machine learning techniques for a more comprehensive analysis. For this previous study, seed images were acquired using a Canon image RUNNER ADVANCE C7270 (Canon Inc, Tokyo, Japan). These images were then analyzed using SmartGrain (version 1.3) high-throughput phenotyping software to extract seed area size (mm²), length (mm), width (mm), length-to-width ratio (LWR), perimeter (mm), circularity (0–1 range, 0: not circular to 1: complete circle), and the distance between the intersection of length & width (IS) and the center of gravity (CG) [29]. Seed darkness and brightness/RGB values (0–255 range) were obtained using a multi-point function in ImageJ version 1.54d [30]. A total of 86,294 seeds were analyzed for seed size-related traits and 25,440 seeds for color-related traits [6]. Any errors produced by the SmartGrain program during image analysis were corrected manually.

To improve normality and homogeneity of variances, all quantitative traits were log-transformed prior to analysis. Pairwise genetic distances, representing genetic distances between taxa, were calculated using publicly available SNP data [6, 28]. The analysis was performed using MEGA 11 software [31]. Distance estimates were generated using the maximum composite likelihood model, a method that considers the probabilities of different types of nucleotide substitutions. To assess the robustness of the distance estimates, variance was estimated using the bootstrap method with 1,000 replicates. The analysis incorporated both transitions and transversions. Rates of nucleotide substitution were assumed to vary among sites and were modeled using a gamma distribution with a shape parameter of 1.00. Pearson’s correlation coefficients (*r*) were calculated to quantify the relationships between genetic distance and the log-transformed seed morphological traits using JMP Pro 17 (SAS Institute Inc., Cary, NC, USA). The significance of the correlations was assessed using a *p*-value threshold of 0.05.

### t-SNE analysis of seed morphology and color

Seed morphology and color data were analyzed using the t-distributed Stochastic Neighbor Embedding (t-SNE) method implemented in the “Multivariate Embedding” platform of JMP Pro 17. t-SNE is a non-linear dimensionality reduction technique that aims to preserve local neighborhood structures in the data when projecting it from a high-dimensional space to a lower-dimensional space, typically two dimensions for visualization [32]. For each analysis (morphology and color separately), the respective quantitative traits were used as input variables. The t-SNE algorithm was configured with the following parameters: output dimensions = 2, perplexity = 30, maximum iterations = 1,000, initial principal component dimensions = 50, convergence criterion = 1e − 8, initial scale = 0.0001, Eta (learning rate) = 200, inflate iterations = 250, and random seed = 123.

### Machine learning-based classification of *Medicago* spp. Based on origin and seed morphology

We utilized the Model Screening platform in JMP Pro 17 to evaluate the performance of eight machine learning models in classifying *Medicago* spp. accessions based on geographic origin (country and region) and seed morphology traits (area, perimeter, length, width, LWR, circularity, and IS & CG). The dataset was randomly divided into training and validation sets (80:20). Models evaluated included Neural Boost (NTanH(3) activation, 20 boosting iterations) [33], Nominal Logistic Fit [34], Support Vector Machines (SVM) (RBF kernel, Cost = 1, Gamma = 0.11111) [35], Bootstrap Forest [36], Generalized Regression Lasso [37], K Nearest Neighbors (*k*-NN) [38], Decision Tree [39], and Naive Bayes [40], with default settings used where not specified. Model performance was assessed using the misclassification rate on the validation set, and feature importance was examined for the top-performing models.

### Machine learning-based classification of *Medicago* spp. Accessions based on seed color and origin

We further evaluated the functionality of seed color and geographic origin for classifying *Medicago* accessions using the Model Screening platform in JMP Pro 17. Eight machine learning models identical to those described in the previous section (Neural Boost, Nominal Logistic Fit, SVM, Bootstrap Forest, Generalized Regression Lasso, *k*-NN, Decision Tree, and Naive Bayes) were trained to predict the species of *Medicago* spp. accessions. Predictor variables included quantitative seed color data (red, green, and blue color intensity values, and brightness) and geographic origin (country). For analyses using only color data, country of origin was excluded as a predictor variable. The dataset was randomly divided into training and validation sets (80% and 20%, respectively). Model performance was evaluated using the misclassification rate on the validation set, with lower rates indicating better performance. Key model settings were consistent with the previous analysis.

### SNP-Based Clustering of *Medicago* spp. Accessions

To investigate the genetic relationships among the *M. sativa* accessions, we performed hierarchical clustering based on SNP genotype data. The SNP data were obtained from a previous study [6] in which GBS was performed on 189 *M. sativa* accessions [28]. Briefly, DNA was extracted from leaf tissue, and GBS libraries were prepared using the ApeKI restriction enzyme [28]. Sequencing was performed on an Illumina HiSeq 2500 platform [28]. Reads were aligned to the *M. truncatula* reference genome (Mt4_0v1), and SNP calling was performed using the TASSEL 5 GBS pipeline [6]. This resulted in a dataset of 8,565 high-quality SNP loci. The SNP data, initially in Variant Call Format (VCF), were converted to a table format using TASSEL 5 [41]. The resulting SNP table was then imported into JMP Pro 17. Hierarchical clustering was performed using the Ward method, which minimizes the within-cluster variance at each step of the clustering process. The resulting dendrogram was used to identify distinct genetic clusters. Additionally, a constellation plot was generated to visualize the relationships among the identified clusters, with distances between clusters reflecting their genetic dissimilarities.

### Genome-Wide association analysis of geographic origin using machine learning

To identify genomic regions associated with the geographic origin of *M. sativa* accessions, we employed a machine learning-based approach analogous to a GWAS. This analysis utilized the previously described SNP genotype data (see section on SNP-based clustering) and focused on predicting the region of origin for *M. sativa* accessions. Regions of origin were categorized as North America, the Middle East, and a combined group representing all other regions, due to the smaller number of samples from these other regions. Three machine learning models were implemented using the Model Screening platform in JMP Pro 17: Boosted Forest, Neural Network, and SVM-RBF. The dataset was randomly partitioned into a training set (80%) and a validation set (20%). Model performance was assessed using the overall classification accuracy in both the training and validation sets. To identify SNPs with the strongest association with geographic origin, we focused on the feature importance scores generated by the Boosted Forest model. Feature importance scores reflect the contribution of each SNP to the model’s prediction accuracy, with higher scores indicating greater importance. The distribution of feature importance scores across the genome was visualized by plotting the log-exponential transformed importance scores against the chromosomal location of each SNP. The top eight SNPs with the highest importance scores were identified and labeled. The genes located in proximity to these top SNPs were identified using the Mtruncatula_Mt4_0v1 genome browser openly available through Phytozome 13 [42] to explore their potential functions and relevance to adaptation.

### Imputation of missing SNP genotypes using machine learning

To address missing data in the SNP dataset, we simulated a machine learning-based approach for imputing hypothetical unknown SNPs. For this analysis, randomly selected accessions (e.g., PI 52243, PI 178981, and PI 467899) were tested to predict SNPs. The random loci, where genotypes were to be predicted, served as the response variables. The remaining SNP loci from other accessions with complete data constituted the predictor variables. Five machine learning models (Decision Tree, Bootstrap Forest, Naive Bayes, Neural Boosted, and SVM-RBF) were trained using the JMP Pro 17 Model Screening option. The dataset was randomly divided into training (80%) and validation (20%) sets, randomly stratified by SNP loci. Model performance was assessed by comparing the predicted genotypes to the withheld true genotypes in the validation set, using overall accuracy as well as precision and recall for each nucleotide. To evaluate the relative weight of different genotypes in predicting the missing SNPs (validation set), feature importance scores were extracted from the Bootstrap Forest model.

## Results

### Correlation between genetic distance and seed morphology traits

**Fig. 1:**
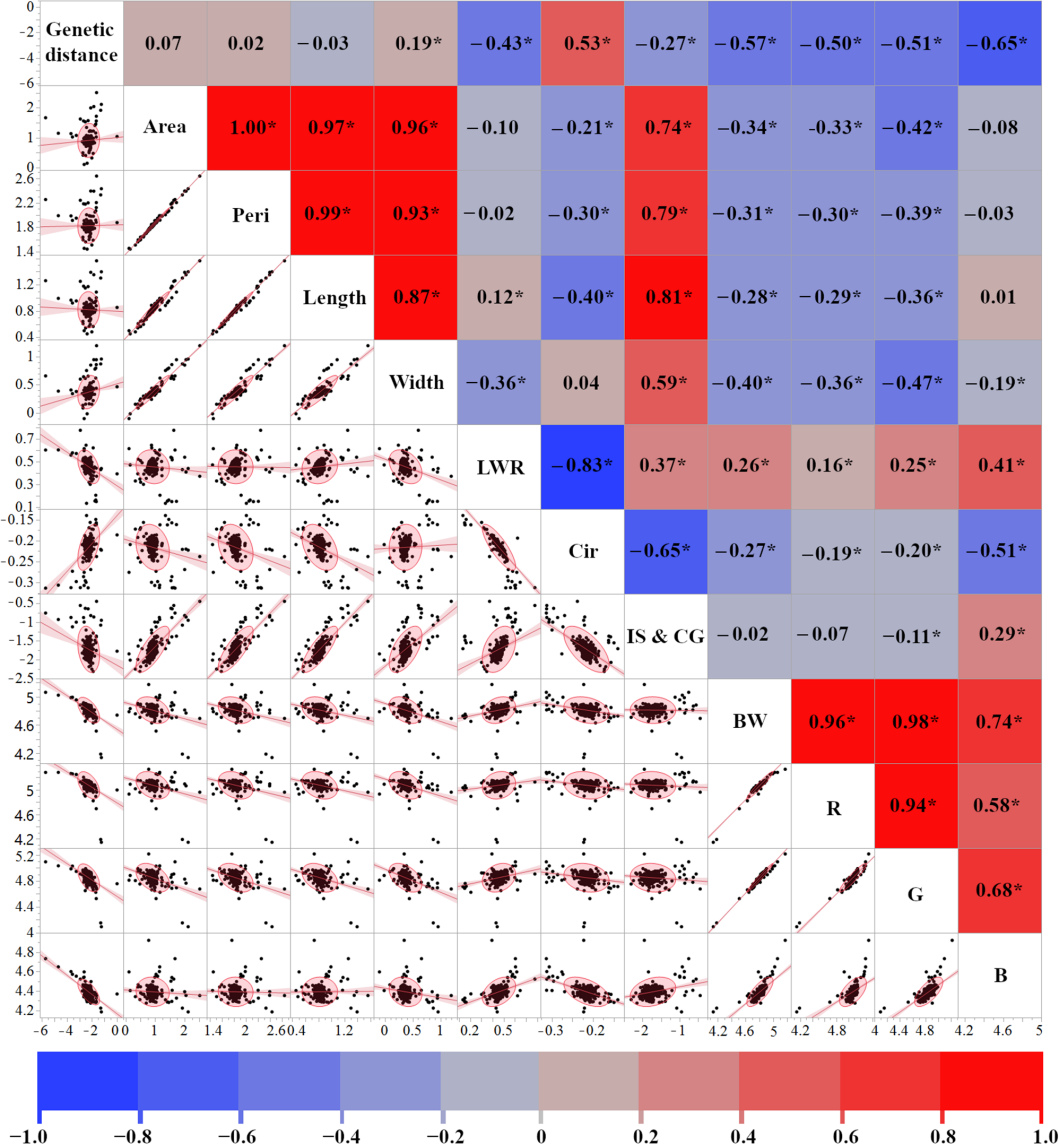
Correlation matrix and scatter plots illustrating the relationships between genetic distance and various seed morphological traits in *Medicago* spp. Morphological traits include: area, perimeter (Peri), length, width, length-to-width ratio (LWR), circularity (Cir), the distance between the intersection of length & width and the center of gravity (IS & CG), black and white balance (BW), red (R), green (G), and blue (B) intensity. The upper triangle of the matrix displays Pearson’s correlation coefficients (*r*), with color intensity representing the strength and direction of the correlation (red for positive, blue for negative). Values marked with an asterisk (*) indicate statistical significance (*p* < 0.05)

To investigate the relationship between genetic relatedness and seed morphology in *Medicago* spp., we performed a correlation analysis using pairwise genetic distances derived from SNP data [6, 28] and a suite of seed morphometric parameters. The results of this analysis are summarized in Fig. 1: Pairwise genetic distances between accessions were calculated using the maximum composite likelihood method implemented in MEGA 11 [31]. We observed a weak positive correlation between genetic distance and seed width (*r* = 0.19, *p* = 0.0007) and a weak negative correlation with the LWR (*r* = − 0.43), and a moderate positive correlation was found between genetic distance and circularity (*r* = 0.53). Interestingly, color intensity parameters, including brightness and red, green, and blue intensities, displayed moderate negative correlations with genetic distance (*r* = − 0.57, − 0.50, − 0.51, and − 0.65, respectively). This suggests a tendency for genetically similar individuals to exhibit more intense seed coloration. Furthermore, IS & CG also showed a weak negative correlation with genetic distance (*r* = − 0.27). Taken together, these findings indicate that seed morphology, particularly traits related to shape and color, are associated with the genetic structure of *Medicago* spp.

### t-SNE visualization of seed morphology and color variation

**Fig. 2:**
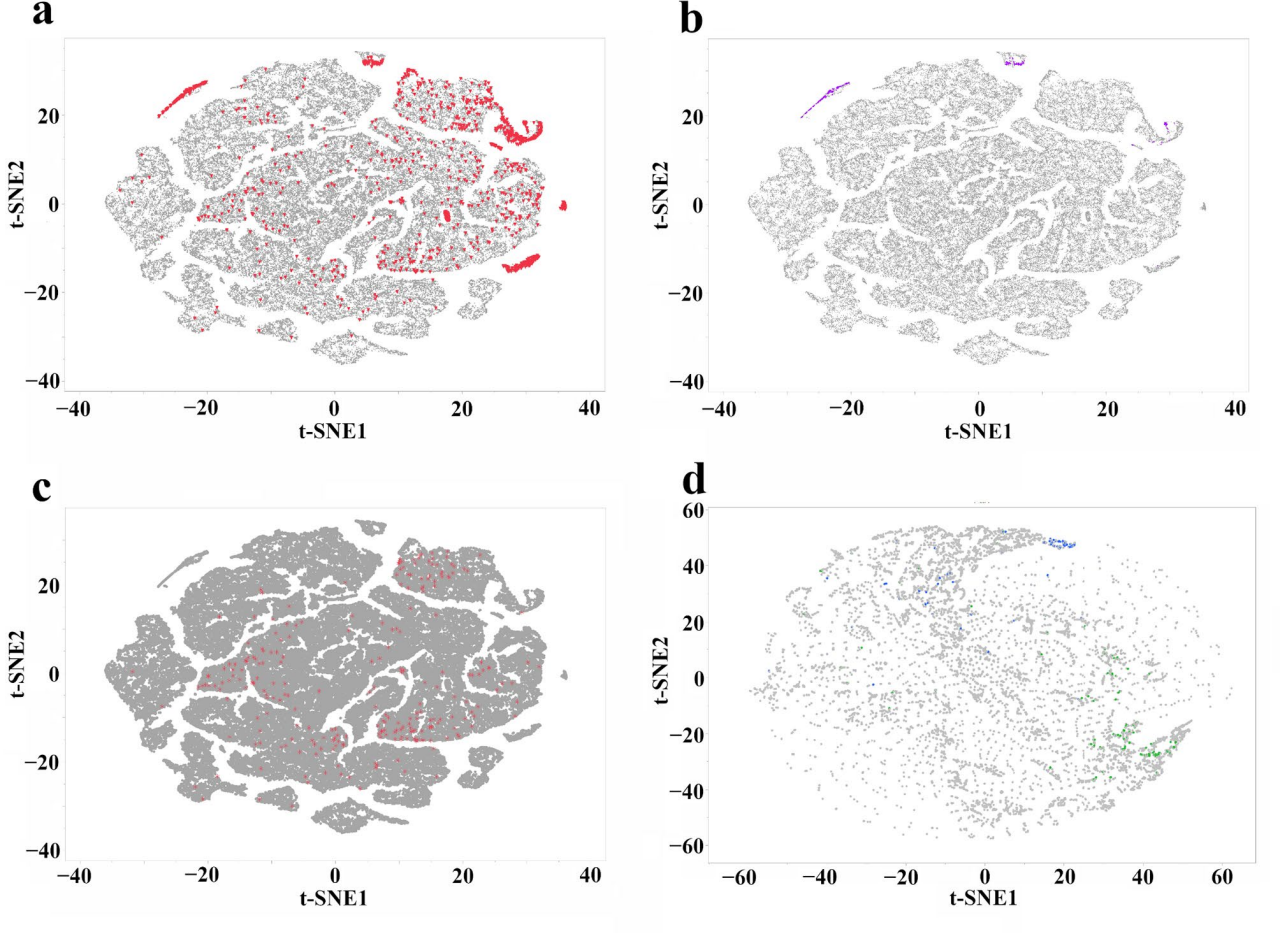
t-distributed Stochastic Neighbor Embedding Visualization of *Medicago* spp. accessions based on seed morphology (**a**-**c**) and color (**d**). (**a**) Accessions from North Africa (Algeria, Morocco, and Tunisia) are highlighted in red, revealing distinct clusters. (**b**) *M. ciliaris* accessions from North Africa, showing two smaller clusters in the upper region (violate). (**c**) *M. sativa* subsp. *sativa* accessions from North Africa, forming dispersed clusters in the middle to upper-right highlighted with * marks. (**d**) Seed color variation, with Algerian accessions in blue and Moroccan accessions in green, demonstrating clear separation despite geographic proximity

To further explore patterns of seed variation, we performed t-SNE analysis on seed morphology and color data (Fig. 2). These seed characteristics can potentially be linked to adaptation to local environmental conditions and reflect underlying genetic differentiation, stressing the value of conserving geographically distinct populations. As shown in Fig. 2a, *Medicago* accessions from North African countries (Algeria, Morocco, and Tunisia) formed distinct red clusters based on seed morphology. This pattern is likely driven by the diverse taxonomic composition of this group, which includes *M. orbicularis*,* M. ciliaris*,* M. marina*,* M. arabica*, and *M. sativa* subsp. *sativa*. Notably, *M. sativa* subsp. *sativa* constituted the majority of the analyzed seeds. The distinct clustering of different *Medicago* spp. based on seed morphology could be useful for species identification and classification, particularly when dealing with closely related taxa.

When focusing solely on *M. ciliaris* (Fig. 2b), two smaller clusters in the upper region of the t-SNE plot became apparent. Even when restricting the analysis to *M. sativa* subsp. *sativa* from North Africa (Fig. 2c), relatively distinct clusters were observed in the middle to upper-right portion of the plot. Furthermore, seed color also exhibited geographic structuring. Despite their geographic proximity, Algerian and Moroccan accessions displayed clear separation in the t-SNE analysis based on seed color (Fig. 2d). For instance, the mean brightness of Algerian seeds (88.73, Standard Error (SE) = 2.16) was significantly lower (darker) than that of Moroccan seeds (138.77, SE = 1.27).

### Performance of machine learning models in classifying *Medicago* spp. Based on Origin and Seed Morphology

Next, we evaluated the performance of various machine learning models in classifying *Medicago* spp. based on their geographic origin (country and region) and seed morphology traits (Table 1). Regions of origin included South America, North America, Europe, Northern Africa, the Middle East, and East and South Asia. Seed morphology traits used as predictors included area, perimeter, length, width, LWR, circularity, and IS & CG. When these combined features were used to predict the geographic origin of *Medicago* spp., most of the tested machine-learning models achieved over 80% accuracy in the validation set. Specifically, Neural Boosted, Nominal Logistic, SVM, Bootstrap Forest, Generalized Regression Lasso, and *k*-NN all performed well, indicating that seed morphology and geographic origin together provide a strong basis for distinguishing *Medicago* spp. populations. This likely reflects underlying genetic and adaptive differences associated with geographic isolation and environmental variation.

**Table 1:** Performance of machine learning models in classifying *Medicago* spp. Based on geographic origin and seed morphology. Models were trained to predict the country and region of origin using seed morphometric data, including area, perimeter, length, width, LWR, circularity, and IS & CG. Misclassification rates are presented for both the training (*n* = 2,158) and the validation sets (*n* = 539). neural boosted, nominal logistic, and SVM-RBF achieved the highest accuracy, with over 85% of accessions correctly classified in both the training and validation sets

Training set		Validation set	
Method	Misclassification rate	Method	Misclassification rate
Nominal Logistic	0.122	Neural Boosted	0.119
Bootstrap Forest	0.071	Nominal Logistic	0.128
Neural Boosted	0.129	Support Vector Machines	0.141
Generalized Regression Lasso	0.148	Bootstrap Forest	0.169
Decision Tree	0.174	Generalized Regression Lasso	0.137
Support Vector Machines	0.144	K Nearest Neighbors	0.165
K Nearest Neighbors	0.172	Decision Tree	0.21
Naive Bayes	0.217	Naive Bayes	0.23

To understand the factors driving the classification performance, we examined the feature importance within each machine learning model (Table 2). neural boosted and nominal logistic fit both identified the country of origin as the most important predictor, hinting at a strong geographic signal in the data. However, the models diverged in their use of region information. neural boosted ranked region as the second most important feature, while nominal logistic fit deemed region redundant after accounting for country, assigning it zero importance. This indicates that neural boosted May be capturing more nuanced, hierarchical geographic patterns not fully represented by country alone. In contrast, SVM prioritized seed morphology traits, with LWR, IS & CG, and length as its top three predictors. Notably, SVM assigned only a little importance to country and region of origin. This emphasis on seed morphology by SVM-RBF aligns with our earlier finding (Fig. 1) that genetic distance is highly associated with seed morphology, suggesting that SVM May be implicitly capturing genetic relationships through these morphological traits. The varying feature importance across models suggests that different algorithms May be learning distinct aspects of the data structure. For instance, neural boosted and nominal logistic fit are likely to capture broader biogeographic patterns associated with country and region, while SVM focuses on finer-scale variation in seed morphology that May reflect both genetic and environmental influences.

**Table 2:** Feature importance in the neural boost, nominal logistic, and SVM-RBF models for *Medicago* spp. Classification based on geographic origin and seed morphology. The table presents the main effect, total effect (where applicable), and importance of each feature in the respective models. For neural boost, “main effect” represents the direct contribution of a feature, while “total effect” includes both direct and indirect contributions via interactions with other features. Importance values are scaled relative to the most important feature within each model. The specific settings used for each model were: neural Boost - NTanH(3)NBoost(20); SVM - RBF kernel with cost = 1 and gamma = 0.11111

Neural Boosted			
Traits	Main effect	Total effect	Importance
Country	0.081	0.78	8
Region	0.07	0.708	7
Length	0.026	0.518	5
Perimeter	0.027	0.518	5
LWR	0.019	0.459	5
Area size	0.015	0.322	3
Circularity	0.01	0.222	2
Width	0.009	0.179	1
IS & CG	0.007	0.167	1
**Nominal Logistic Fit**			
**Traits**	**Main effect**	**Importance**	***P*** **-value**
Country	106582.9	22	0
Length	3.226	1	0.00059
Area size	1.836	1	0.01459
Circularity	0.861	0	0.13763
Perimeter	0.267	0	0.54106
Width	0.261	0	0.54859
LWR	0.129	0	0.743
IS & CG	0.02	0	0.95462
Region	0	0	1.0
**Support Vector Machines**			
**Traits**	**Main effect**	**Total effect**	**Importance**
LWR	0.021	0.873	9
IS & CG	0.013	0.819	9
Length	0.011	0.717	7
Circularity	0.005	0.684	7
Perimeter	0.009	0.634	6
Width	0.005	0.357	3
Area size	0.003	0.263	2
Region	0.001	0.057	0
Origin	0.001	0.047	0

### Predicting Seed Size in *Medicago* spp. Using Machine Learning

Larger seeds generally possess more significant nutrient reserves, leading to faster initial growth, better establishment, and a higher probability of survival, particularly under stressful conditions [43–45]. Therefore, the ability to accurately predict seed size based on easily measurable characteristics is of considerable agronomic value. To this end, we evaluated the performance of various machine learning models in predicting seed area in *Medicago* accessions using geographic origin, *Medicago* spp. information, and seed shape-related traits (LWR, circularity, and IS & CG) as predictors (Table 3). We excluded direct measures of seed dimensions (length, width, and perimeter) to assess the predictive power of shape, taxonomic information, and origin information alone. Remarkably, both Boosted Tree and Bootstrap Forest models achieved over 80% accuracy (R-squared = 0.807 and 0.804, respectively) in the validation set, demonstrating the strong predictive capacity of these models. This high accuracy is particularly noteworthy because it implies that seed shape characteristics and taxonomic information, in combination with geographic origin, can serve as effective proxies for overall seed size. This finding has important implications for high-throughput phenotyping, as it suggests that seed size can be estimated indirectly from shape features extracted from images, potentially circumventing the need for direct size measurements.

**Table 3:** Performance of machine learning models in predicting seed area in *Medicago* spp. Based on geographic origin, species, and seed shape. Models were trained to predict seed area using country of origin, region of origin, *Medicago* species, LWR, circularity, and IS & CG. The table presents the R-squared values for both the training (*n* = 23,659) and the validation sets (*n* = 5,915). boosted tree and bootstrap forest achieved R-squared values greater than 0.80 in the validation set, demonstrating strong predictive accuracy using only geographic origin, species, and shape-related traits

Training set		Validation set	
Method	R-Square	Method	R-Square
Bootstrap Forest	0.826	Boosted Tree	0.807
Boosted Tree	0.824	Bootstrap Forest	0.804
Decision Tree	0.798	Neural Boosted	0.797
Neural Boosted	0.793	Decision Tree	0.795
Fit Least Squares	0.773	K Nearest Neighbors	0.786
Fit Stepwise	0.773	Fit Stepwise	0.782
Generalized Regression Lasso	0.773	Fit Least Squares	0.782
K Nearest Neighbors	0.771	Generalized Regression Lasso	0.782

**Fig. 3:**
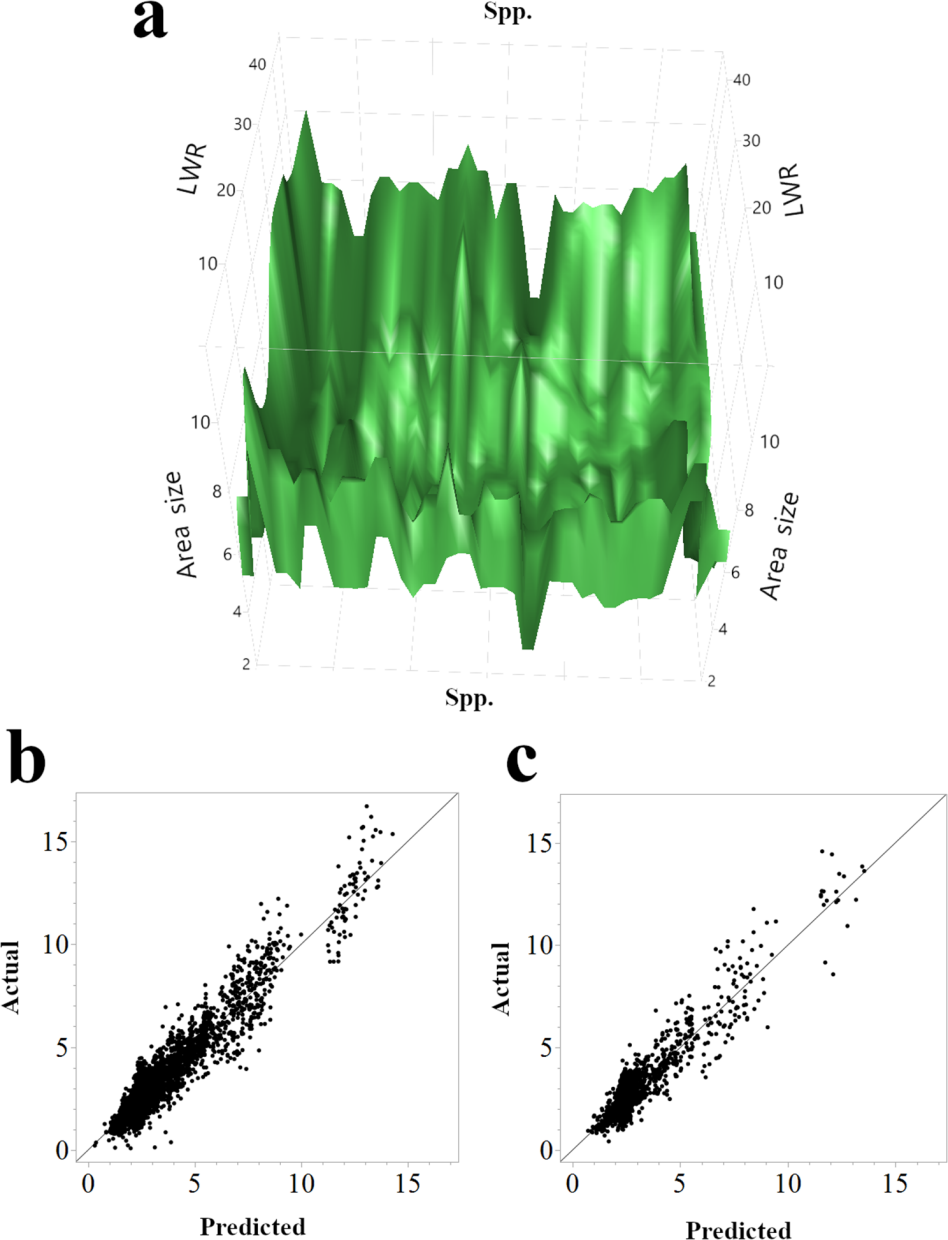
Relationships between *Medicago* spp., seed shape, and seed area size, and the accuracy of seed area prediction. (**a**) A representative three-dimensional surface plot illustrating the combined influence of *Medicago* spp., LWR, and area size from the Neural Boosted model. (**b**) Scatter plot comparing actual and predicted seed area sizes from the Boosted Tree model in the training set. (**c**) Scatter plot comparing actual and predicted seed area sizes from the Bootstrap Forest model in the validation set

To gain insight into the factors driving the accurate prediction of seed area, we examined the feature importance within the top-performing models: boosted tree, bootstrap forest, and neural boosted (Table 4). Across all three models, *Medicago* spp. Was identified as the most important predictor, accentuating the strong influence of taxonomic classification on seed size. This finding aligns with the concept that seed size is a key functional trait reflecting adaptations to various environmental conditions and dispersal strategies [46]. Different species within the *Medicago* genus have likely evolved distinct seed morphologies in response to selective pressures such as risk reduction, escape from crowding, and escape from Sib competition, as theorized by venable and brown [46]. This relationship is further visualized in Fig. 3a, which depicts the interaction between species, seed shape (LWR), and seed area. For boosted tree and bootstrap forest, the country of origin Was the second most important predictor, demonstrating that geographic factors conceivably acting as proxies for environmental or genetic variation also play a considerable role in determining seed area. Interestingly, in the neural boosted model, the second most important feature Was IS & CG, followed closely by LWR and country. The prominence of shape descriptors in the neural boosted model suggests that this model might be capturing complex, potentially non-linear relationships between seed shape and size that are not as readily detected by the other models. The predictive performance of the boosted tree and bootstrap forest models is further illustrated in Fig. 3b and C, which are presented in scatter plots of actual versus predicted seed area for each model. In all cases, region information Was less critical for predicting seed size due to redundancy. Taken together, these results emphasize the noteworthy role of species identity in determining seed area in *Medicago* spp., while also displaying the contribution of geographic origin and subtle shape variations captured by seed shape (IS & CG and LWR).

**Table 4:** Feature importance in the boosted tree, bootstrap forest, and neural boosted models for predicting seed area size in *Medicago* spp. Direct measures of seed size (length, width, and perimeter) were excluded from this analysis. The table presents metrics of feature importance, which include the number of splits, sum of squares, portion, main effect, total effect, and importance score, depending on the specific model. These metrics reflect the contribution of each feature to the model’s predictive accuracy. Importance values are scaled relative to the most important feature within each model

Boosted Tree			
Traits	Number of splits	Sum of square	Portion
spp.	185	62072.488	0.891
Country	136	3503.615	0.05
IS & CG	247	2356.041	0.034
LWR	193	1040.461	0.015
Circularity	166	568.873	0.0082
Region	27	92.488	0.0013
**Bootstrap Forest**			
**Traits**	**Number of splits**	**Sum of square**	**Portion**
spp.	768	10345.068	0.774
Country	1394	2049.079	0.153
IS & CG	5950	477.473	0.0357
LWR	5913	286.909	0.0215
Circularity	5704	146.269	0.0109
Region	336	62.855	0.0047
**Neural Boosted**			
**Traits**	**Main effect**	**Total effect**	**Importance**
spp.	0.238	0.381	4
IS & CG	0.179	0.326	3
LWR	0.111	0.287	3
Country	0.151	0.278	3
Region	0.078	0.163	1
Circularity	0.008	0.015	0

### Classification of *Medicago* spp. Based on Seed Color and Geographic Origin

To further investigate the factors influencing the classification of *Medicago* spp., we evaluated the performance of eight machine learning models using seed color data (red, green, blue intensity, and brightness) and geographic origin (country) as predictors (Table 5). Initially, when models were trained using only color-related variables, the classification accuracy reached approximately 60% for Neural Boosted, SVM-RBF, and Generalized Regression Lasso in the validation set (data not shown). Considering the presence of 29 different *Medicago* spp. in the dataset, this level of accuracy is substantially higher than random chance (approximately 3.45%), suggesting that seed color alone carries crucial information for distinguishing between different *Medicago* spp. However, when geographic origin (country) was incorporated as an additional predictor, the classification accuracy improved dramatically, reaching nearly 80% for several models in the validation set. Neural Boosted achieved the highest accuracy (Table 5), closely followed by Nominal Logistic, Generalized Regression Lasso, and SVM. The importance of the color variables in the Neural Boosted model is further detailed in Table 6. While country of origin was the most important predictor (Main effect = 0.416, Importance = 10), red, blue, green, and brightness also made significant contributions (Table 6), insinuating that subtle variations in seed color, as captured by these variables, provide additional information for classifying *Medicago* species beyond geographic origin alone.

**Fig. 4:**
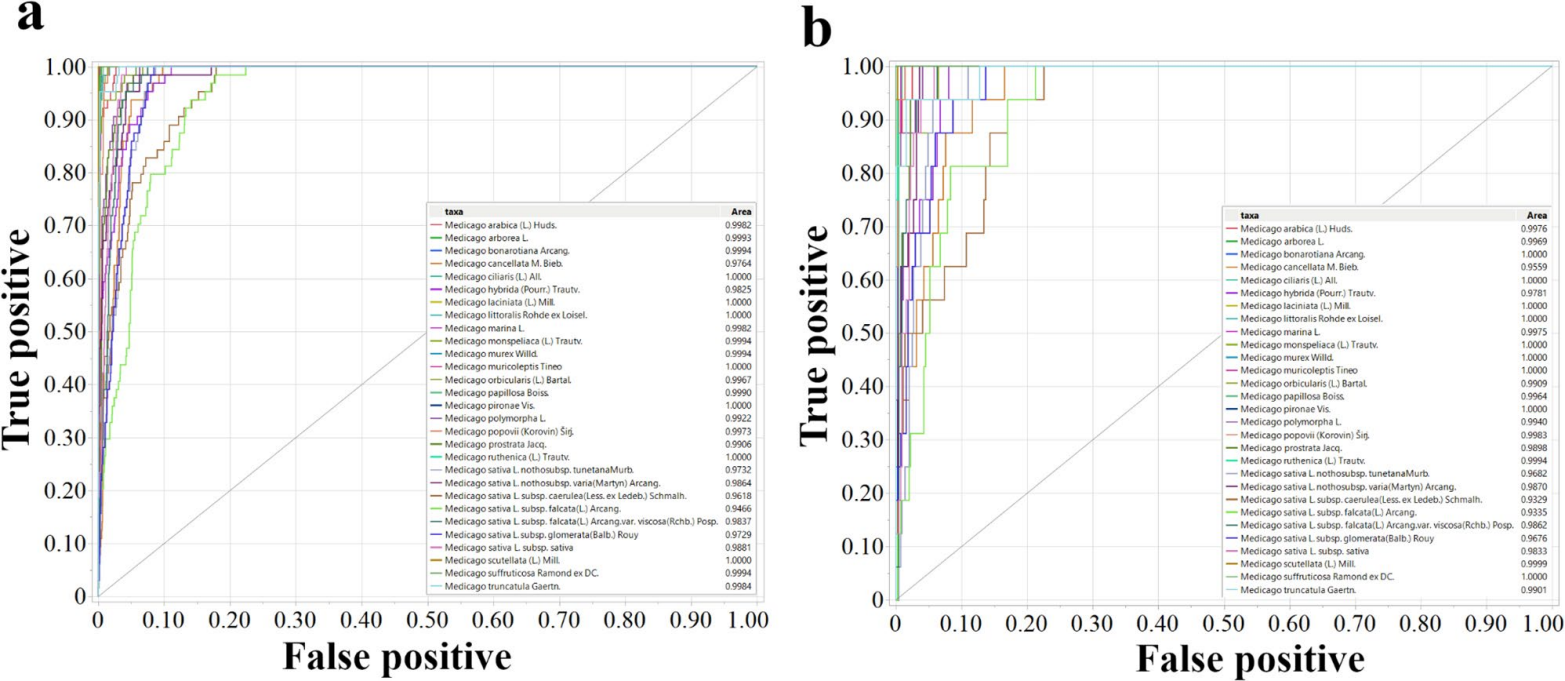
Receiver operating characteristic curves for classifying *Medicago* species using the Neural Boosted model with color and geographic origin as predictors. (**a**) ROC curves for the training set. (**b**) ROC curves for the validation set. Each ROC curve represents the performance of the model in classifying a specific *Medicago* species

The performance of the Neural Boosted model is further illustrated in Fig. 4, which displays the Receiver Operating Characteristic (ROC) curves for each *Medicago* species in both the training and validation sets. The ROC curves demonstrate the trade-off between the true positive rate and the false positive rate for different classification thresholds. The area under each ROC curve (AUC) provides a measure of the model’s overall performance in classifying a given species, with an AUC of 1 representing perfect classification and an AUC of 0.5 representing random chance. As shown in Fig. 4, the Neural Boosted model achieved high AUC values for most species, demonstrating strong overall classification performance. However, the model’s accuracy varied across different taxa. Species such as *M. bonarotiana*, *M. ciliaris*, and *M. murex* were classified with near-perfect accuracy (AUC close to 1), while *M. sativa* subsp. *caerulea* had a notably lower AUC value of 0.933, signaling greater challenges in distinguishing this subspecies based on the predictors used. This substantial improvement in accuracy upon the addition of geographic origin implies a strong association between seed color and the geographic location of *Medicago sativa* populations. The variation in model performance across different species, as revealed by the ROC curves, suggests that some species possess more distinctive combinations of seed color and geographic origin, while others exhibit greater overlap in these traits, making them more challenging to classify.

**Table 5:** Performance of machine learning models in classifying *Medicago* spp. Based on geographic origin (country) and seed color (red, green, blue intensity, and brightness). The table presents the misclassification rates for each model in both the training set (*n* = 1,856) and the validation set (*n* = 464)

Training set		Validation set	
Method	Misclassification rate	Method	Misclassification rate
Bootstrap Forest	0.104	Neural Boosted	0.21
Neural Boosted	0.184	Nominal Logistic	0.213
Nominal Logistic	0.195	Generalized Regression Lasso	0.22
Generalized Regression Lasso	0.2	Support Vector Machines	0.237
Support Vector Machines	0.209	Bootstrap Forest	0.259
Decision Tree	0.272	Decision Tree	0.295
K Nearest Neighbors	0.251	K Nearest Neighbors	0.252
Naive Bayes	0.316	Naive Bayes	0.341

**Table 6:** Feature importance in the neural boosted model for *Medicago* spp. Classification based on color and origin. The table presents the main effect, total effect, and relative importance of each predictor in the model

Neural Boosted			
Traits	Main effect	Total effect	Importance
Country	0.416	0.95	10
Red	0.02	0.286	3
Blue	0.021	0.269	2
Green	0.021	0.252	2
Brightness	0.013	0.163	1

### Genetic relationships among *Medicago* accessions revealed by SNP clustering

**Fig. 5:**
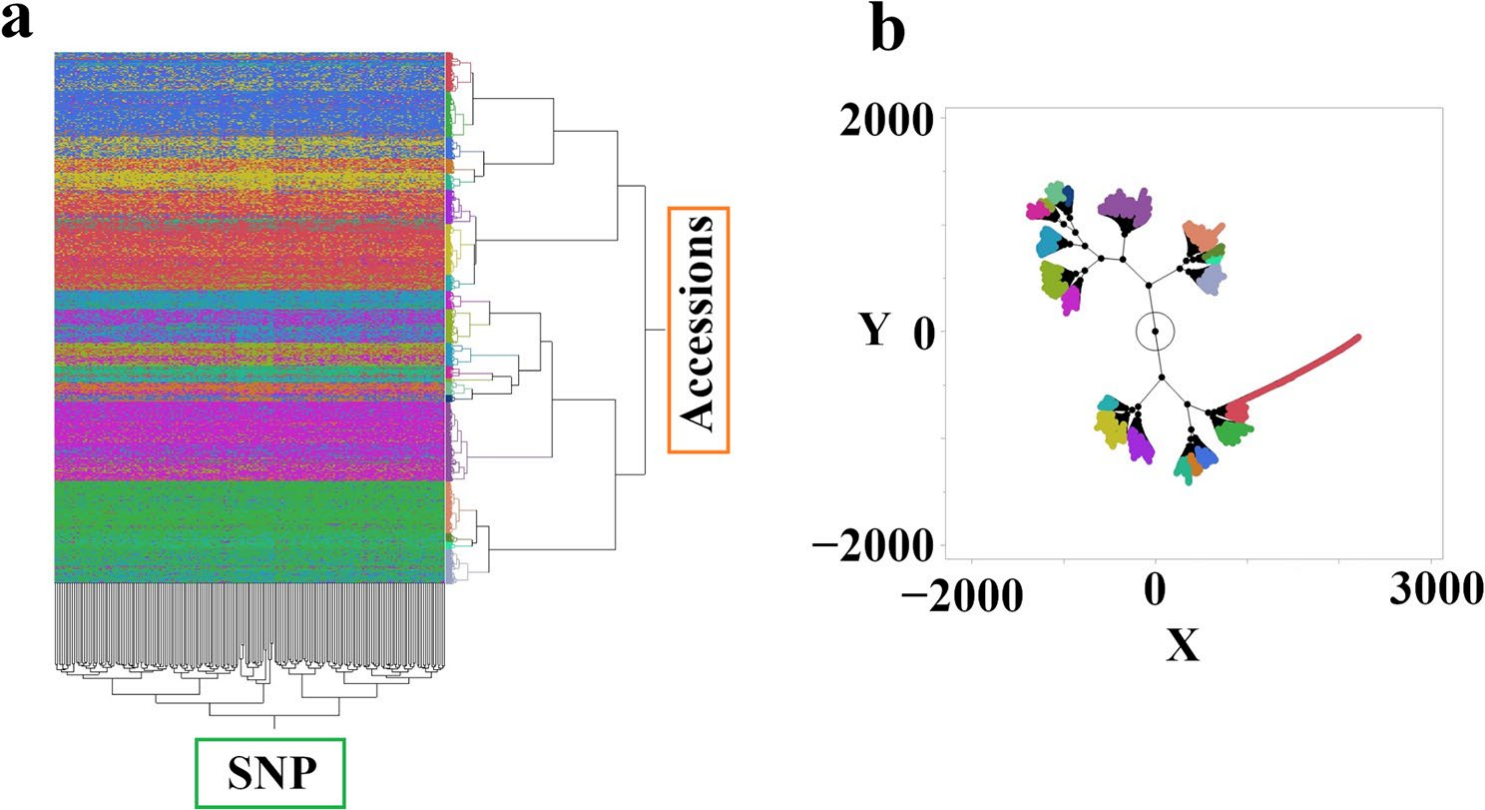
Hierarchical clustering and constellation plot of *Medicago* accessions based on SNP data. (**a**) Hierarchical cluster dendrogram of 189 *Medicago* accessions genotyped at 8,565 SNP loci. Clustering was performed using the Ward method. Branches represent individual accessions (x-axis) and SNP loci (y-axis), while the colors represent the 20 distinct clusters identified. (**b**) Constellation plot visualizing relationships among the 20 SNP-based clusters. The plot is based on the same distance matrix used for the hierarchical clustering. Each node represents a cluster, and the length of the links indicate the degree of relatedness, with shorter links representing closer relationships

To investigate genetic relationships among the accessions, we performed hierarchical clustering on 182 *M. sativa* accessions, for which we had both SNP genotype data (8,565 loci) and phenotypic data. These accessions were a subset of the 318 used in the phenotypic analyses. While our SNP dataset genotyped 189 accessions in total, only 182 had both data types available. Using the Ward method, we identified 20 distinct clusters based on the similarity of their SNP genotype profiles (Fig. 5a). The relationships among these clusters, visualized in a constellation plot (Fig. 5b), reflect their overall genetic similarity, with shorter distances indicating closer relationships.

### Genome-Wide association analysis of geographic origin using machine learning

**Fig. 6:**
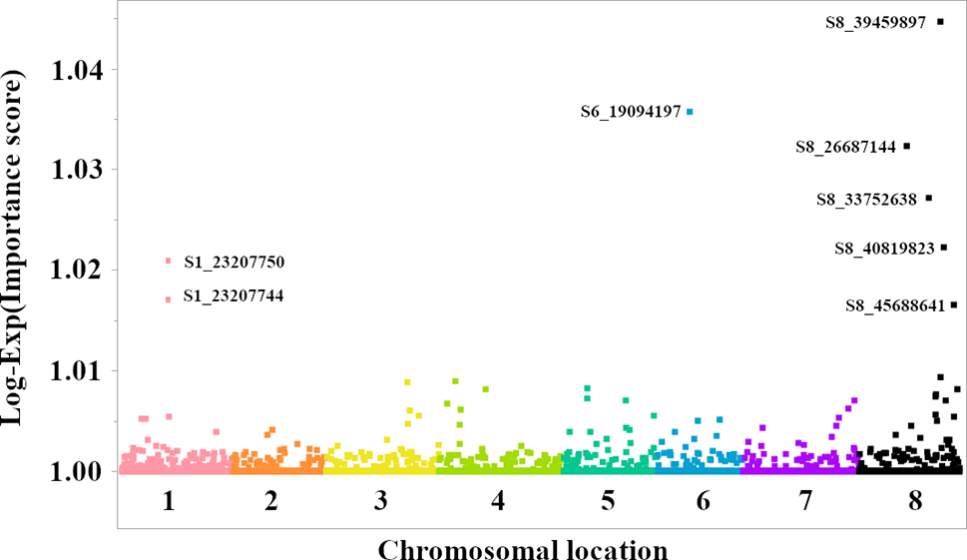
Genome-wide feature importance scores from a Boosted Forest model predicting the region of origin of *Medicago* spp. accessions. The X-axis represents chromosomal locations of SNPs, while the Y-axis shows the log-exponential transformed importance scores, log(Exp[Importance score]). These scores reflect each SNP’s contribution to predicting the region of origin. SNPs are color-coded by chromosome, and the top eight SNPs with the highest scores are labeled

The SNP-based clustering (Fig. 5) provided initial insights into the genetic structure of the *Medicago* collection, producing 20 distinct clusters and implying plausible associations with geographic origin and/or phenotypic traits. To further explore this connection and identify specific genomic regions associated with geographic origin, we performed a GWA analysis using a machine learning approach. Specifically, we employed the Boosted Forest, Neural Network, and SVM-RBF models to predict the geographic origin of *M. sativa* (alfalfa) accessions based on their SNP genotypes, focusing on North America, the Middle East, and a combined group of other regions due to smaller sample sizes in the latter. The models achieved over 90% accuracy in the training set and over 85% accuracy in the validation set, indicating a strong association between SNP genotypes and geographic origin in *M. sativa*. This analysis revealed several SNPs with notably high importance scores, suggesting their pronounced contribution to distinguishing between *M. sativa* accessions from different regions. Based on the Boosted Forest model, the most important SNP was S8_39459897, followed by S6_19094197, S8_26687144, S8_33752638, and S1_23207750 (Fig. 6). The prominence of SNPs located on chromosomes 8, 6, and 1 (S8, S6, and S1, respectively) suggests that these genomic regions may harbor genes or regulatory elements that have played a role in the adaptation of *M. sativa* to different environments.

### Imputation of missing SNP genotypes using machine learning

To explore the potential for imputing missing SNP genotype information in *Medicago* accessions, we employed a machine learning approach to predict the genotype at specific SNP loci based on the genotypes at other loci across the genome. This approach is conceptually similar to imputation, where missing data are inferred from patterns observed in the available data. To simulate missing data and evaluate imputation accuracy, we randomly selected SNP loci across the genome to be treated as the response variable (e.g., PI 52243, PI 178981, and PI 467899), while the remaining SNP loci from all accessions served as predictor variables. The dataset was split into training (80%) and validation (20%) sets. We evaluated the performance of five machine learning models: Decision Tree, Bootstrap Forest, Naive Bayes, Neural Boosted, and SVM.

**Fig. 7:**
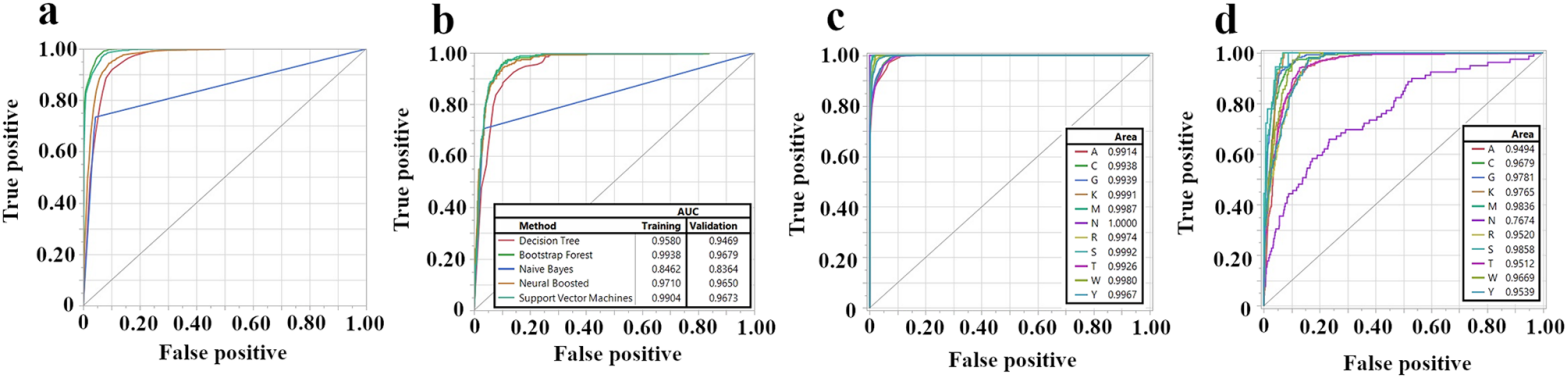
Performance of machine learning models in predicting SNP genotypes in *Medicago sativa* accessions. (**a**) ROC curves for the training set using the Bootstrap Forest model to predict SNP genotypes of PI 467,899, a representative accession. (**b**) ROC curves for the validation set for PI 467,899 using the Bootstrap Forest model. (**c**) ROC curves for the training set showing individual nucleotide prediction accuracy using the Bootstrap Forest model, with each curve representing the accuracy of predicting a specific nucleotide (A, C, G, T, W, Y, K, M, N, R, or S). (**d**) ROC curves for validation set showing individual nucleotide prediction using the Bootstrap Forest model. The models were trained using SNP data from other accessions to predict genotypes at specific loci in accessions with missing data. The AUC is presented in the inset table, with higher AUC values indicating better prediction accuracy. The results demonstrate the potential of machine learning for imputing missing SNP genotypes and highlight the variation in prediction accuracy across different nucleotides

As shown in Fig. 7a and b, which depict the ROC curves for the training and validation sets of PI 467,899, a representative accession, using Bootstrap Forest model. The models achieved high accuracy in predicting the missing SNP genotypes, often exceeding 70% for many of the tested SNP loci using the bootstrap forest model. For instance, in the case of PI 467,899, the Bootstrap Forest model achieved an accuracy of 70.4%. While all models performed nearly identically to the Bootstrap Forest model, Naive Bayes consistently underperformed compared to the other methods.

When examining the accuracy of prediction for individual nucleotides (Fig. 6c and d), we found that the models performed exceptionally well for the known nucleotides (A, T, C, G), often achieving over 80% accuracy (e.g., for PI 467899: A = 0.808, C = 0.855, G = 0.893, T = 0.834 in the validation set, using the Bootstrap Forest model). In contrast, ambiguous nucleotide codes (e.g., K, M) and the undetermined code “N” showed significantly lower accuracy (K = 0.32, M = 0.413, *R* = 0.43, and *N* = 0). Notably, the model mostly predicted “N” as one of the four known nucleotides (A, T, C, or G), suggesting that these “N” calls might represent true nucleotides that were not confidently called during sequencing or initial data processing. These results were consistent across multiple randomly selected SNP loci. The overall accuracy of approximately 70% may be partially attributed to the difficulty in predicting these ambiguous or undetermined nucleotides, particularly “N,” having an accuracy rate = 0.

To assess whether the models were relying on a small number of highly informative genotypes or utilizing broader patterns across multiple genotypes, we examined the feature importance scores from the Bootstrap Forest model for PI 467,899. The analysis revealed that while the model used information from 46 genotypes in total to predict the unknown SNP, a small subset of genotypes contributed disproportionately to the prediction accuracy. For example, the SNP information from PI 467,921 had the highest importance score (18), followed by PI 468,014 and PI 467,958 (both with importance scores of 10). This implies that the genotypes of certain accessions are particularly informative for predicting the genotypes of others, possibly reflecting close evolutionary relationships or shared ancestry.

## Discussion

This study provides a comprehensive analysis of seed morphology, color, and geographic origin in a diverse collection of *Medicago* spp. accessions. We found that seed size, shape, and color exhibited substantial variability across the accessions. Seed size in particular was established as an agronomically important trait due to its association with seedling vigor and establishment success, a key factor in early growth and establishment, and could contribute to more accurate yield estimations and improved breeding strategies [11].

Our machine learning models, including Neural Boosted, Bootstrap Forest, SVM-RBF, and others, successfully classified *Medicago* spp. accessions based on a combination of geographic origin and seed morphology, achieving over 80% accuracy in several cases. Furthermore, seed color alone allowed for classification accuracy significantly higher than random chance, indicating that this trait carries considerable information about the identity of *Medicago* spp. The inclusion of geographic origin, specifically the country of origin, dramatically improved classification accuracy, highlighting the strong geographic structuring of seed morphology and color variation in *Medicago* spp. These initial findings reiterate the major role of both genetic and environmental factors in shaping seed characteristics and demonstrate the potential of using these traits, along with geographic information, for effective germplasm characterization.

To further explore the genetic underpinnings of the observed phenotypic variation and investigate the association between genotype and geographic origin, we conducted a hierarchical clustering analysis based on over 8,500 SNP markers genotyped across 189 *Medicago* accessions. This analysis revealed 20 distinct genetic clusters, suggesting a substantial population structure within the collection. Investigating whether these genetic clusters exhibit any enrichment for specific seed morphologies or geographic origins could offer further evidence for the role of these factors in shaping the population structure of *M. sativa*. The relationships among these clusters, visualized through a constellation plot, provide insights into the genetic relatedness among accessions and potentially reflect patterns of historical gene flow or adaptation to different environments. Importantly, these SNP-based clusters provide a framework for investigating the genetic basis of the observed phenotypic variation in seed traits and geographic origins. We hypothesized that specific genetic clusters might be enriched for accessions from particular geographic regions or with distinct seed characteristics.

To test this hypothesis and identify specific genomic regions associated with geographic origin, we performed a GWAS using machine learning models (i.e. Boosted Forest, Neural Network and SVM-RBF) to predict the geographic origin of *M. sativa* accessions based on their SNP genotypes. This analysis divulged a strong association between SNP genotypes and origin, with the models achieving high accuracy in both training and validation sets.

By examining the feature importance scores from the Boosted Forest model, we identified several SNPs with significantly high importance, particularly on chromosomes 1, 6, and 8.

Intriguingly, SNP S8_39459897, which was identified as the most important predictor, is located directly within the coding region of the gene *MtrunA17Chr8g0378361*. This gene encodes a putative carbohydrate-binding X8 domain-containing protein. The X8 domain is found in a variety of proteins across different species, often associated with binding to complex carbohydrates like cellulose and xylan [47]. In plants, such proteins play roles in cell wall modification, cell signaling, and defense responses [48]. Variations in this gene could therefore affect cell wall integrity or signaling pathways related to stress response. Similarly, SNP S6_19094197 is located nearby a region annotated as both a hypothetical protein-coding gene (*MtrunA17Chr6g0469391*) and a transposable element (*MtrunA17_Chr6R0109300*). The proximity of a SNP to both a hypothetical gene and a transposable element (TE) requires careful consideration. TEs are known to influence gene expression and can contribute to phenotypic variation [49]. Accordingly, the SNP may affect the hypothetical protein’s function or alternatively, it might influence the activity of the TE, which in turn could affect the expression of nearby genes. SNP S1_23207750 is located close to a gene encoding a Kunitz-P Trypsin Inhibitor (*MtrunA17Chr1g0173431*) and is within the coordinates of transposable element (*MtrunA17_Chr1R0123740*). Kunitz-type trypsin inhibitors are involved in plant defense mechanisms aimed primarily against herbivores and pathogens by inhibiting the activity of digestive enzymes [50, 51]. Variations in this gene might influence the plant’s ability to defend itself against biotic stresses. However, the presence of the TE (*MtrunA17_Chr1R0123740*) in the same region adds another layer of complexity. The presence of these geographically informative SNPs in or near genes with functions related to stress response, genome stability, and gene regulation, along with their association with TEs, implies that selection pressures associated with different environments have shaped the genetic diversity of *M. sativa*, perhaps leading to local adaptation. Further functional studies are required to elucidate the precise mechanisms by which these SNPs, the associated genes, and the neighboring TEs contribute to stress tolerance variation in plants.

A key aspect of this study was our application of machine learning to impute missing SNP genotype information in *M. sativa* accessions. While numerous methods exist for missing value imputation, recent reviews highlight the growing prominence of machine learning approaches [52]. Previous studies have explored the use of machine learning for genotype imputation in various species, such as rice [53], but our approach offers specific advantages by leveraging machine learning to not just impute missing data but also to illuminate the strong linkage disequilibrium (LD) patterns and genomic architecture of *M. sativa*. For instance, Islam et al. [53] developed a deep learning model called AGIC, which uses a stacked autoencoder to impute missing values and compress genome-wide polymorphism data in rice. While their focus was on data compression and imputation using a different model architecture, their work demonstrates the power of deep learning, particularly autoencoders, for handling genomic data with missing values [53]. The imputation process, in effect, becomes a tool for exploring the genomic landscape of this important forage crop. By training models, specifically the Bootstrap Forest model, on a subset of loci with complete data, we predicted genotypes at loci with missing data with often over 70% accuracy. The models performed exceptionally well in predicting the four standard nucleotides (A, T, C, G), achieving over 80% accuracy across randomly selected loci. The overall accuracy, however, was influenced by the difficulty in predicting ambiguous or undetermined nucleotide calls (e.g., “N”), suggesting that many of these “N” calls in the original dataset may represent true nucleotides that were not confidently called during sequencing or data processing. Despite this challenge, the high accuracy for standard nucleotides demonstrates the capabilities of our method. As noted by Thomas and Rajabi [52], the performance of imputation techniques can vary depending on the characteristics of the dataset and the type of missingness. Our results, focusing on SNP data in *M. sativa*, contribute to the understanding of how machine learning can be effectively applied in plant genomics. The ability to impute missing data with such accuracy has noteworthy implications for the utilization of germplasm resources, allowing for the inclusion of accessions that might otherwise be excluded due to incomplete data, effectively “rescuing” potentially valuable genetic diversity.

Furthermore, our analysis of feature importance revealed that a relatively small number of highly informative genotypes can be sufficient for accurate prediction. This finding further supports the existence of strong LD patterns within the *M. sativa* genome, although the extent of LD can vary depending on the specific population and markers used. For instance, Han et al. [54] investigated LD in diploid alfalfa using both Simple Sequence Repeat (SSR) markers and candidate gene sequences, finding very limited LD among SSR markers genome-wide but observing that within-gene LD decayed below an *r*^2^ of 0.2 within 300 bp in three of four candidate genes. In our previous study, we found that the mean linkage disequilibrium has been shown to extend to less than 5 kilobases [6]. This relatively rapid decay of LD, while not directly comparable to our tetraploid data, underscores the influence of considering population-specific LD patterns when designing and interpreting association studies, as well as selecting appropriate markers in downstream analysis, such as imputation.

This understanding could guide future, more targeted genotyping efforts. For instance, focusing on a subset of key accessions could enable efficient imputation for a much larger collection, maximizing the value of existing genomic data and facilitating downstream analyses such as GWAS and genomic selection. Notably, our method’s success with a relatively simple dataset and its focus on *M. sativa*, coupled with the interpretability offered by the Bootstrap Forest model and the relatively high accuracy in comparison to other methods, distinguishes it from previous imputation approaches and exhibits the capability it possesses for broader application in plant genomics. Further research could explore the integration of different machine learning approaches, such as combining the strengths of autoencoders and tree-based models, to further enhance imputation accuracy and genomic insights in *M. sativa* and other crop species.

## Conclusion

In conclusion, this study demonstrates the power of integrating phenotypic, genotypic, and geographic data to unravel the diversity patterns within *Medicago* spp. and establishes the crucial role of seed characteristics in this important forage crop. Our analysis revealed substantial variation in seed morphology and color, identified distinct genetic clusters, and pinpointed genomic regions potentially associated with geographic adaptation, providing valuable insights for germplasm characterization and conservation. Our findings regarding the influence of seed size are consistent with the broader need for accurate yield prediction in alfalfa, as expressed by Whitmire et al. [55], who demonstrated the effectiveness of machine learning in predicting alfalfa biomass. Similarly, our ability to predict seed size, a key factor in early growth and establishment, could contribute to more accurate yield estimations and improved breeding strategies.

Furthermore, our SNP-based clustering uncovered distinct genetic groups within *Medicago*, which hints at the gravity of conserving this diversity. The GWAS analysis identified specific genomic regions associated with geographic origin, providing candidate genes involved in cell wall integrity and stress response (*MtrunA17Chr8g0378361*), herbivore and pathogen resistance (*MtrunA17Chr1g0173431*), and genome stability (*MtrunA17Chr6g0469391*) that may contribute to local adaptation. These findings provide a foundation for future investigations into the functional significance of these genes.

Crucially, our novel machine learning-based imputation method successfully addressed the challenge of missing data, enhancing the quality and completeness of our genomic dataset. The imputation process also illuminated strong linkage disequilibrium patterns within the *M. sativa* genome, affirming the need to consider LD in future genomic studies. The high accuracy of our imputation, particularly for the four standard nucleotides, demonstrates this approach’s capability to maximize the usefulness of existing germplasm resources and facilitate more powerful downstream analyses, such as GWAS and genomic selection.

Future research should focus on validating the imputation method using independent datasets and experimentally confirming the predicted genotypes. Additionally, exploring the functional significance of the identified genetic clusters and their association with specific phenotypic traits through functional studies and further validation of these GWAS results will be crucial. Ultimately, a deeper understanding of the genetic and environmental determinants of seed characteristics in *Medicago* spp. will contribute to the development of improved varieties with enhanced adaptability, stress tolerance, and yield potential, while also informing strategies for the effective conservation of valuable germplasm resources.

### Data Availability

The raw phenotype and SNP data used in this study are available in Botkin et al. (2024) 10.1038/s41598-024-67790-4. The machine learning-related data generated and analyzed during the current study are available from the corresponding author on reasonable request.

## Data Availability

The raw phenotype and SNP data used in this study are available in Botkin et al. (2024) https://doi.org/10.1038/s41598-024-67790-4. The machine learning-related data generated and analyzed during the current study are available from the corresponding author on reasonable request.
